# Vitamin D Supplementation: A Potential Approach for Coronavirus/COVID-19 Therapeutics?

**DOI:** 10.3389/fimmu.2020.01523

**Published:** 2020-06-23

**Authors:** John F. Arboleda, Silvio Urcuqui-Inchima

**Affiliations:** ^1^Group of Immunovirology, Faculty of Medicine, University of Antioquia, Medellin, Colombia; ^2^Behavioural Science and Health Care Habits Unit, Comfama, Medellin, Colombia

**Keywords:** SARS-Cov-2, COVID-19, vitamin D, antiviral, anti-inflammatory

The ongoing pandemic: Coronavirus disease 2019 (COVID-19) caused by SARS-CoV-2 has become one of the most important epidemiological events within the last 100 years, causing devastating consequences for the public health systems and the socioeconomical tissue around the world (1–3). Infection with SARS-CoV-2 can lead to a mild or highly acute respiratory syndrome fueled by altered secretion of inflammatory cytokines (cytokine storm) that can be fatal within children, elderly populations, patients with chronic pulmonary or hypertension diseases, and people living in cities with poor air quality (3, 4). While viral spreading and severity indexes are growing as the virus reaches new geographic areas, clinical trials for several vaccine prospects are being performed with the caveat that it may take more than 6 months to provide data of their efficiency and sero-protection levels (2, 5–7). Consequently, the remaining alternatives to counteract COVID-19 disease and pandemics are currently based on (i) the implementation of a broad-spectrum of antivirals that could attenuate the virus infection, (ii) clinical relief of acute inflammatory symptoms, and (iii) social isolation of *at risk* populations to avoid propagation (5, 8). However, given the uncertainty for specific treatment and the economic consequences of social isolation, especially in developing countries, repurposing of current drugs it is imperative to develop quick, and cost-effective therapeutic strategies to protect vulnerable populations (9).

A potential alternative is vitamin D, a natural immunoregulator that has been demonstrated to enhance antimicrobial activity against several pathogens including respiratory viruses (10, 11). Indeed, both *in vitro* observations and supplementation trials have extensively shown the restrictive features of vitamin D against respiratory viruses including: syncytial virus, influenza, and coronaviruses (8, 10–19) and other non-respiratory viruses, such as human immunodeficiency virus 1, hepatitis c virus, and dengue virus (20–22). Classically, the mechanisms reported to support these antiviral effects are based on the ability of vitamin D to upregulate antimicrobial peptides and induce antiviral cytokines to interfere the viral replicative cycle (10, 12, 23–31). Interestingly, we have recently reported a novel molecular vitamin D-derived mechanism that can also target early stages of the viral cycle via downregulating the expression of host cell receptors for viral attachment. This novel mechanism is responsible for impairing binding and entry of dengue virus, thus, restricting *in vitro* infection (22) and likely, further dissemination to other primary host cells.

SARS-CoV-2 can target both upper and lower epithelial lung cells and gain access to, via anchoring of its spike (S) protein to angiotensin-converting enzyme 2 (ACE2) receptor (32–36). This receptor is an important enzyme for the regulation of the Renin-Angiotensin System (RAS) which regulates blood pressure and vascular balance. Notably, ACE2 is highly expressed in patients with hypertension, diabetes mellitus, coronary heart diseases, and cerebrovascular disease, which could explain the higher risk of severe and fatal COVID-19 within these patients (37, 38). In fact, recently it has been demonstrated that SARS-CoV-2 can also bind and infect central nervous system cells through targeting the ACE2 receptor, implicating participation of this neurotropic mechanism into the disease severity and mortality (39).

As concerning inferences may arise from all these observations, it is important to note that ACE2 receptor has been broadly known to be downregulated by vitamin D activity (40). Mechanistically, vitamin D works as a potent negative endocrine regulator of the RAS via the canonical vitamin D receptor pathway which can suppress RAS and downregulates the expression of ACE2 both *in vitro* and *in vivo* (37, 41). Indeed, it has been documented that vitamin D-derived suppression of RAS can be elicited via vitamin D inhibition of CREB (cAMP response element-binding protein), a transcription factor key for the renin gene regulation (42). Moreover, these experimental observations have been corroborated by mounting clinical and epidemiologic evidence, where decreased serum levels of vitamin D have been correlated with increased activity of RAS, higher plasma renin activity, and high blood pressure levels (43–46). For instance, improved vitamin D serum concentrations due to oral supplementation within hypertensive patients that were previously vitamin D insufficient, were associated with improvement in the control of blood pressure (47).

In light of these observations, we anticipate in this comment that the regulating effects of vitamin D on the renin-angiotensin system, specifically, on ACE2 receptor downregulation could contribute with restriction of SARS-CoV-2, similarly to what we have reported with dengue virus (22). Accordingly, an increasing number of studies are postulating blockade of this receptor as a likely therapeutic strategy for COVID-19 (2, 48–50). Furthermore, besides infection, severity of COVID-19 is strongly associated with altered and prolonged pro-inflammatory responses in the lung, that ultimately lead to abnormal respiratory events and further organ failure (3). In line with literature, our experimental model has shown that beyond the vitamin D-derived downregulation of relevant receptors for viral attachment, this hormone can also contribute with fine tuning of the altered pro-inflammatory responses induced by the virus (22, 51). In fact, others have reported that vitamin D-derived alleviation of pulmonary damage, caused by inflammation, in a model of acute lung injury, and respiratory distress was related to modulation of several members of RAS, including ACE2 receptor (37, 40, 41, 52).

In line with findings from other reports (10), our observations that vitamin D-derived antiviral mechanisms can restrict viral infection and attenuate the pro-inflammatory response (22) have been corroborated *ex vivo* in two different vitamin D supplementation exploratory studies. We demonstrated that a daily oral supplement of 4000 IU of vitamin D during 10 days represented an adequate dose to enhance dengue virus control and reduce the cytokine response, *in vitro*, suggesting that vitamin D status can, in fact, restrict the viral assault (53, 54). Accordingly, several studies have highlighted the beneficial role of vitamin D sufficiency levels and supplementation for viral respiratory infections (55–57). Indeed, outbreaks and higher incidence of respiratory viruses such as influenza and coronavirus are common beyond subtropical areas with low sunlight exposure levels and prevalence of vitamin D deficiency/insufficiency such as Europe and Northern United States, which have been highly affected by COVID-19 (10, 11, 58).

While several drugs targeting the ACE2-dependet entry pathway for SARS-CoV-2 still await for validation and assessment of their side effects (6, 7, 49, 59), at least three clinical trials aimed to elucidate the protective role of vitamin D role on COVID-19 disease severity are currently progressing in Spain, France and United States (60–62). Moreover, a mounting number of observations worldwide, are consistently suggesting the preventive and prophylactic features vitamin D status for COVID-19 mortality (63–66).

Our hypothesis provides a call for research pathways to unravel the role of vitamin D on the pathogenesis of COVID-19, but beyond that, it also opens a hope window for a more immediate, accessible, natural, and cost-effective strategy to prevent, treat and ameliorate propagation of SARS-CoV-2. In summary, we postulate that conventional oral vitamin D supplementation can be a readily strategy to aim: (i) restriction of SARS-CoV-2 infection via downregulation of ACE2 receptor, and (ii) attenuation of disease severity by down-tuning the pulmonary pro-inflammatory response or cytokine storm that fuels COVID-19 severity. Therefore, verifying its beneficial role by means of epidemiologic, clinical and experimental *in vivo* and *in vitro* evidence may turn Vitamin D into a new “*at hand tool*” to protect vulnerable populations and mitigate the impact of the current pandemic events, especially in countries with reduced capability of their public health systems.

## Author Contributions

All authors listed have made a substantial, direct and intellectual contribution to the work, and approved it for publication.

## Conflict of Interest

The authors declare that the research was conducted in the absence of any commercial or financial relationships that could be construed as a potential conflict of interest.
